# Can the Neutrophil to Lymphocyte Ratio Be Used to Determine Gastric Cancer Treatment Outcomes? A Systematic Review and Meta-Analysis

**DOI:** 10.1155/2016/7862469

**Published:** 2016-01-26

**Authors:** Jingxu Sun, Xiaowan Chen, Peng Gao, Yongxi Song, Xuanzhang Huang, Yuchong Yang, Junhua Zhao, Bin Ma, Xinghua Gao, Zhenning Wang

**Affiliations:** ^1^Department of Surgical Oncology and General Surgery, First Hospital of China Medical University, Shenyang 110001, China; ^2^Department of Dermatology, First Hospital of China Medical University, Shenyang 110001, China

## Abstract

The prognostic role of neutrophil to lymphocyte ratio (NLR) in gastric cancer remains controversial. We aimed to quantify the prognostic role of peripheral blood NLR in gastric cancer. A literature search was conducted in PubMed, EMBASE, and Cochrane databases. The results for overall survival (OS) and progression-free survival (PFS)/disease-free survival (DFS) are expressed as hazard ratios (HRs) with 95% confidence intervals (CIs). 19 studies with 5431 patients were eligible for final analysis. Elevated NLRs were associated with a significantly poor outcome for OS (HR = 1.98; 95% CI: 1.75–2.24, *p* < 0.001) and PFS (HR = 1.58; 95% CI: 1.32–1.88, *p* < 0.001) compared with patients who had normal NLRs. The NLR was higher for patients with late-stage compared with early-stage gastric cancer (OR = 2.76; 95% CI: 1.36–5.61, *p* = 0.005). NLR lost its predictive role for patients with stage IV gastric cancer who received palliative surgery (HR = 1.73; 95% CI: 0.85–3.54, *p* = 0.13). Our results also indicated that prognoses might be influenced by the NLR cutoff values. In conclusion, elevated pretreatment NLRs are associated with poor outcome for patients with gastric cancer. The ability to use the NLR to evaluate the status of patients may be used in the future for personalized cancer care.

## 1. Introduction

Gastric cancer is the second most common cause of cancer mortality worldwide, in part because most patients are diagnosed with advanced, inoperable disease [1]. Early detection, surgical resection, and adjuvant therapy have improved the survival of patients with early-stage gastric cancer. Even for patients with advanced gastric cancer who receive potentially curative resections, the 5-year survival remains at still 30–50% [2]. In addition, many patients experience side effects from surgery and adjuvant therapy [3, 4]. Treatment strategies are determined by TNM staging system. However, many patients of the same TNM stage have different prognoses [5]. It is important to identify factors that predict the treatment response and survival of gastric cancer patients.

Recently, an increasing number of studies have focused on tumor microenvironment, which is associated with the systemic inflammatory response and may play an important role in cancer tumorigenesis and progression [6, 7]. Many markers of systemic inflammation response to tumors have been investigated as prognostic and predictive biomarkers, such as C-reactive protein (CRP) and erythrocyte sedimentation rate (ESR) [8, 9]. The neutrophil-to-lymphocyte ratio (NLR) is a potential inflammation-based prognostic indicator for several types of cancer, such as renal cell carcinoma [10], hepatocellular carcinoma [11], and colorectal carcinoma [12]. Some studies have indicated that elevation in the NLR for patients with gastric cancer may predict worse prognosis [13]. However, other studies [14] have shown no such association. The association between the NLR and clinicopathological characteristics and prognosis function of patients with gastric cancer remains unclear.

In this study, we conducted a systematic review and meta-analysis to quantify the prognostic role of the peripheral blood NLR in gastric cancer. We also aimed to determine the correlation between the NLR and clinicopathological factors for patients with gastric cancer.

## 2. Materials and Methods

### 2.1. Systematic Search Strategy

This study was performed in accordance with the Preferred Reporting Items for Systematic Reviews and Meta-Analysis (PRISMA) guidelines [15]. A sensitive search strategy was developed for all English-language literature published before November 2014 using PubMed, EMBASE, and the Cochrane Database of Systematic Reviews. The search strategy included the keywords “neutrophils”, “lymphocytes”, “neutrophil-to-lymphocyte ratio”, “NLR”, and “stomach neoplasms”. Review articles and bibliographies of other relevant articles were individually examined to identify additional studies.

### 2.2. Inclusion and Exclusion Criteria

All of the studies included were comparative studies of patients with gastric cancer who had a high or low peripheral blood NLR. Treatments included curative surgery, palliative resection, or palliative chemotherapy. The hazard ratio (HR) and 95% confidence intervals (CIs) or survival curves for overall survival (OS), progression-free survival (PFS), or disease-free survival (DFS) were required. Articles lacking full text and data that could not be acquired from the authors were excluded. When multiple studies were reported by the same team from the same institute and were performed at the same time, only the latest article or the one with the largest data set was included.

### 2.3. Data Extraction and Quality Assessment

Data collection and analyses were performed by two researchers using predefined tables, which included author, publication time, sample size, age, treatment, follow-up, tumor differentiation, TNM stage, tumor size, and cutoff value used to define the elevated NLR, OS, PFS, and DFS. If a study did not provide a HR for the OS, PFS, or DFS, we used Engauge Digitizer version 4.1 to distinguish survival curves and calculate HRs and 95% CIs. The first reviewer (Jingxu Sun) extracted data and another reviewer (Xiaowan Chen) checked the data with any disagreements resolved by discussion and consensus.

Two reviewers (Jingxu Sun and Xiaowan Chen) performed quality assessment of the observational studies using the Newcastle-Ottawa scale [16]. Articles with NOS scores ≥6 were considered to be of high quality because standard validated criteria for important end points have not been established. The mean value for all included articles was 6.1 and the details are shown in Table 2.

### 2.4. Statistical Analysis

Meta-analysis was performed with Review Manage version 5.2 (Cochrane Collaboration, Copenhagen, Denmark) and Stata version 12.0 (Stata, College Station, TX, USA), and Microsoft Excel 2010 (Microsoft, Santa Rosa, CA, USA) was used for statistical analysis. If there was any disagreement, discussion among the authors was required. The HRs and 95% CIs for available data were calculated to identify potential associations with the OS, PFS, or DFS in two groups, using the method reported by Tierney et al. [17]. The odds ratios (ORs) and 95% CIs were calculated as effective values of the results of the analysis between NLR and clinicopathological characteristics. Statistical heterogeneity among studies was quantified using the *χ* ^2^ and *I* ^2^ statistic. The *I* ^2^ statistic was derived from the *Q* statistic ([*Q* − df/*Q*] × 100), and it provides a measure of the proportion of the overall variation attributable to heterogeneity among studies. If the heterogeneity test was statistically significant, then the random effects model was used. The source of heterogeneity was investigated by meta-regression and subgroup analysis. The *p* value threshold for statistical significance was set at 0.05 for effect sizes. Publication bias was analyzed by Begg's test and Egger's bias indicator test, and the results were then expressed in a funnel plot.

## 3. Results

### 3.1. Studies Included and Methodological Quality

**Figure 1:**
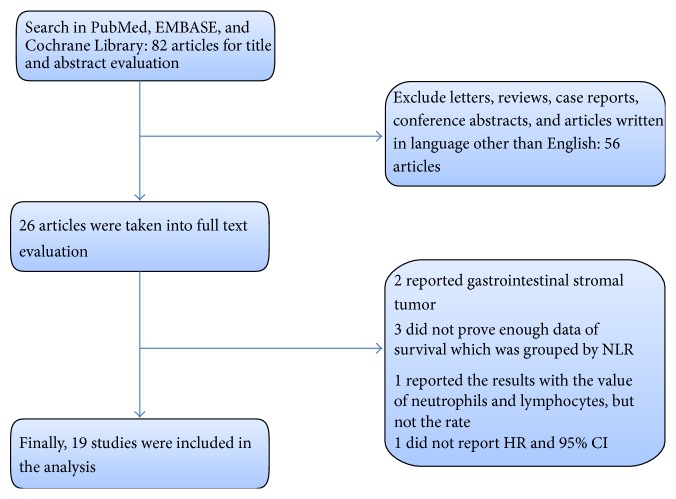
PRISMA flow diagram for the meta-analysis.

The initial search strategy identified 82 articles, including 26 that were further evaluated after initial review of the titles and abstracts. After further consideration of the remaining articles, 19 studies [13, 14, 18–34] involving 5431 patients were included in our meta-analysis. All of the included articles were observational cohort studies and all of the NLRs were tested before treatment. A flowchart of the search strategy is shown in Figure 1. The study characteristics are summarized in Table 1. Six were studies were from Japan, six were from Korea, three were from China, two were from Italy, one was from Turkey, and one was from Egypt. Ten of these articles had <200 patients and another nine had >200 patients. All of the included articles provided the TNM stage of patients, and four only studied patients in stage IV. The NOS score was summarized in Table 2.

### 3.2. OS and NLR for Patients with Gastric Cancer

**Figure 2:**
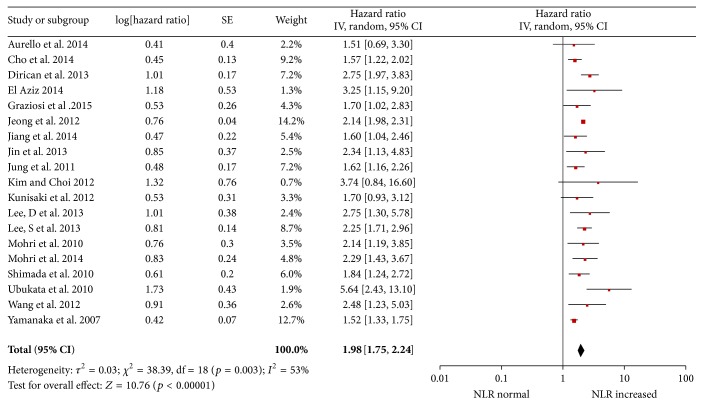
Hazard ratiofor overall survival.

Survival was significantly longer for patients with a low NLR than those with a high NLR with a pooled HR of 1.98 (95% CI: 1.75–2.24, *p* < 0.001; Figure 2) and the heterogeneity was significant (*p* = 0.003, *I* ^2^ = 53%).

We performed meta-regression and subgroup analysis to explore heterogeneity by country, year of publication, sample size, cut-off value for NLR, and whether patients underwent surgery. Almost all of the subgroup analyses had no influence on the heterogeneity of the pooled analysis with the exception of the subgroup distinguished by sample size (Table 3). Meta-regression also demonstrated that sample size may explain the source of heterogeneity (*p* = 0.021).

### 3.3. PFS, DFS, and NLR for Patients with Gastric Cancer

**Figure 3:**
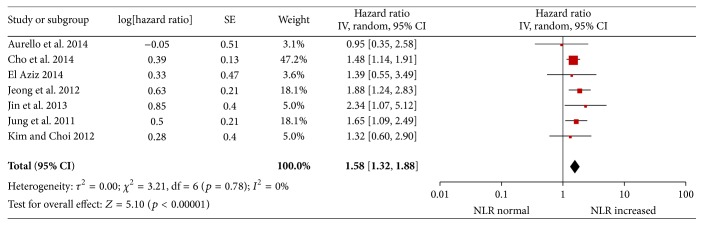
Hazard ratio for disease-free survival.

There were four studies [20, 22, 25, 29] that reported a correlation between the PFS and NLR, and three studies [21, 28, 30] provided data regarding DFS and NLR. The pooled results show that patients with an elevated NLR have shorter PFS and DFS after treatment compared with patients with a normal NLR (HR = 1.58; 95% CI: 1.32–1.88, *p* < 0.001; Figure 3). There was no evidence of statistical heterogeneity (*p* = 0.78, *I* ^2^ = 0%). For PFS, the pooled HR was 1.61 (95% CI: 1.31–1.97, *p* < 0.001) with no significant heterogeneity. For DFS, the pooled HR was 1.48 (95% CI: 1.05–2.09, *p* < 0.001) with no significant heterogeneity.

### 3.4. TNM Stage and NLR of Patients with Gastric Cancer

Four studies [13, 19, 24, 31] reported data on the TNM stage and NLR for patients with gastric cancer. We classified TMN stage I/II in one group and stage III/IV to another group to evaluate the role of NLR. The pooled OR produced by a random-effect model was 2.76 (95% CI: 1.36–5.61, *p* = 0.005), and the significant heterogeneity was observed (*p* = 0.002, *I* ^2^ = 80%; Table 3). Patients with higher NLR tended to have advanced gastric cancer.

### 3.5. NLR for Patients with Stage III and IV Gastric Cancer

Five studies [13, 14, 26, 30, 32] reported the NLR and OS of patients with stage III gastric cancer. Elevated NLR was associated with worse outcome (HR = 2.17; 95% CI: 1.67–2.83, *p* < 0.001), and there was no significant heterogeneity (*p* = 0.29, *I* ^2^ = 20%).

Seven studies [18, 20, 26, 29, 30, 32, 34] reported NLR for patients with stage IV gastric cancer. Two of these studies [30, 32] provided data about patients who received palliative gastrectomy with or without metastasis resection. Three studies [20, 30, 34] reported patients with stage IV gastric cancer who underwent palliative treatment. For patients with stage IV gastric cancer, high NLRs were associated with poor prognosis (HR = 1.81; 95% CI: 1.50–2.18, *p* < 0.001). We performed subgroup analysis to determine whether the NLR could be a marker for different treatments such as resection or palliative chemotherapy. Patients who underwent resection had a HR of 1.73 (95% CI: 0.85–3.54, *p* = 0.13), and patients who received palliative chemotherapy had a HR of 1.83 (95% CI: 1.49–2.24, *p* < 0.001). All of the above results are shown in Table 3.

### 3.6. Tumor Differentiation and the NLR of Patients with Gastric Cancer

Three studies [13, 20, 30] reported the level of tumor differentiation and the NLR in gastric cancer. The combined OR was 1.05 (95% CI: 0.77–1.43, *p* = 0.75; Table 3) with no heterogeneity (*p* = 0.38, *I* ^2^ = 0%), and the pooled results indicated that there was no correlation between tumor differentiation and NLR for patients with gastric cancer.

### 3.7. Carcinoembryonic Antigen (CEA) and NLR for Patients with Gastric Cancer

Two studies [23, 24] have presented data on the CEA level and NLR for patients with gastric cancer. There was no significant correlation between CEA and NLR for gastric cancer patients, with an OR of 1.43 (95% CI: 0.64–3.21, *p* = 0.37; Table 3).

### 3.8. Cutoff Value for the NLR for Patients with Gastric Cancer

All of the studies reported cutoff values for the NLR. We collected all the cutoff values for the NLR and divided the studies into four groups based on the quartiles of their cutoff values. The three quartiles were as follows: 2.20, 3.00, and 4.00. The HR in Subgroup 1 (cutoff value of NLR < 2.20) was 1.80 (95% CI: 1.43–2.26, *p* < 0.001), 1.88 in Subgroup 2 (2.20 ⩽ cutoff value of NLR < 3.00; 95% CI: 1.56–2.26, *p* < 0.001), 2.31 in Subgroup 3 (3.00 ⩽ cutoff value of NLR < 4.00; 95% CI: 1.81–2.94, *p* < 0.001), and 2.36 in Subgroup 4 (cutoff value of NLR ⩾ 4.00; 95% CI: 1.38–4.03, *p* < 0.001; Table 3).

### 3.9. Publication Bias

**Figure 4:**
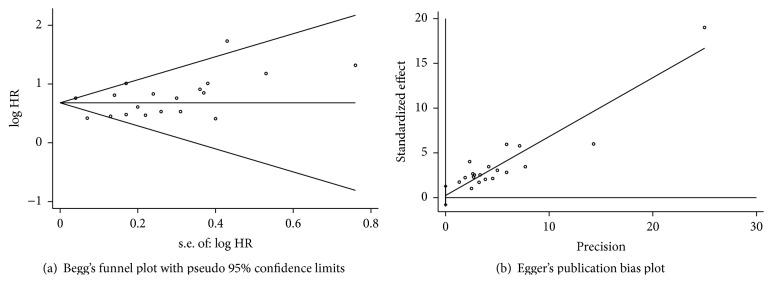
(a) Begg's test. (b) Egger's test.

Publication bias was demonstrated using Begg's funnel plot and Egger's test. Begg's funnel plot demonstrated that there was no publication bias for OS (*p* = 0.141, Figure 4(a)). Egger's test also showed that there was no publication bias for OS (*p* = 0.628, Figure 4(b)).

## 4. Discussion

Several studies have suggested that elevated NLR, an inflammation-based prognostic score, is correlated with the poor survival of many types of cancers. The mechanism of NLR responses to tumors may be explained as an increase in neutrophils or decrease in lymphocytes that may restrain lymphokine-activated killer cells and increase metastasis [35]. However, some other studies have reported negative results for the NLR for prognosis and clinicopathologic characteristics. At the same time, the optimal cutoff value for the NLR is uncertain. For gastric cancer in particular—a disease which has been proved to be associated with chronic inflammation—the conclusions remain controversial. To address the questions above, we performed this study using meta-analysis.

We included 19 articles with 5431 patients with gastric cancer to evaluate the prognostic role of NLR. We found that pretreatment NLR can predict OS and PFS for patients with gastric cancer. We also investigated the relationship between the cutoff values and predictive function of NLR in gastric cancer and found a trend that the NLR might influence prognosis along with the increase of cutoff value. Moreover, we used subgroup and meta-regression analysis to establish the source of heterogeneity, and subgroup analysis found lower heterogeneity in each group, as expected. The results indicated that elevated NLR was associated with late stages of gastric cancer, and elevated NLR predicted poor prognosis for patients who received palliative chemotherapy for stage IV gastric cancer.

In recent decades, our understanding of the inflammatory microenvironment of cancer has improved, and research has focused on the association between cancer and inflammation. Inflammation plays an important role in the development and progression of several cancers by suppressing or stimulating tumor cells [36]. Therefore, many inflammatory indicators, including NLR, platelet to lymphocyte ratio, or CRP, are diagnostic and prognostic biomarkers for various cancers [37]. NLR, in particular, is a prognostic indicator for several other solid cancers such as urinary [38] and colorectal [39, 40] cancer. Chronic inflammation may be caused by* Helicobacter pylori,* and it is an important risk factor for stomach neoplasms [41]. In our meta-analysis, we demonstrated that the prognosis of patients with high NLRs was worse than that for patients with a normal NLR amongst early-stage gastric cancers. Furthermore, we found that high NLRs are associated with late-stage gastric cancer. However, the mechanisms involved in the association of elevated NLR and poor outcome for patients with gastric cancer remain unclear. There are several explanations for the correlation between poorer prognosis and elevated NLR in gastric cancer. A high NLR reflects a decrease in the number of lymphocytes and/or an elevated number of neutrophils. Neutrophils may play an important role in cancer development and progression by offering a suitable microenvironment for their growth. Circulating neutrophils may contain and secrete the majority of circulating vascular endothelial growth factor, interleukin-18, and matrix metalloproteinase, which are thought to be closely associated with tumorigenesis, development, and metastasis [42–44]. Furthermore, the antitumor immune responses of activated T cells and natural killer cells may be inhibited by an elevated number of neutrophils surrounding tumor tissues. Therefore, a high level of circulating neutrophils may have a negative effect on patients with gastric cancer and lead to poor outcome. At the same time, lymphocytes play an important role in cellular adaptive immunity against cancer by attacking and clearing tumor cells at the outset of tumorigenesis [45]. Patients who have lymphocyte infiltration surrounding their tumors may have a better prognosis than those with less or no infiltration [46]. In addition, lymphocytes may be suppressed by large numbers of neutrophils when two cells are cocultured [47]. Our results indicate that an elevated NLR denotes a pretreatment inflammatory condition that is correlated with poor prognosis for patients with gastric cancer. Although the NLRs were tested before treatment and status of patients was favorable, NRL still might be influenced by a number of confounding factors in peripheral blood. So the control of confounding factors in studies about the association between NLR and gastric cancer may be an important research point in the future.

For most gastric cancer patients, recurrence and metastasis remain the main factors that may cause death and influence survival, even after curative resection [48]. The identification of sensitive markers that can predict prognosis and help select patients who may receive different treatments is needed. TNM staging is a good indicator for gastric cancer patients who undergo surgery [21]. Inflammation-based prognostic scores such as NLR could predict the prognosis of patients before they receive treatment. In this study, we analyzed the relationship between the NLR level and TNM stage in gastric cancer. Elevated NLR was associated with late-stage gastric cancer and indicated that elevated NLR indicates worse prognosis. We analyzed the predictive role of NLR for patients with stage III/IV gastric cancer. Elevated NLR predicts poor outcome for patients with stage III/IV gastric cancer. Furthermore, immunosuppression induced by surgery is associated with delaying postoperative recovery time, increasing the cancer recurrence rate, and reducing the survival time [49]. We analyzed NLR in stage IV gastric cancer to establish whether pretreatment NLR values indicate prognosis for patients who have received surgery. Elevated NLR indicated poor outcome for patients with stage IV gastric cancer. Nevertheless, subgroup showed that elevated NLR was associated with poor outcome in stage IV gastric cancer patients who received palliative chemotherapy and the surgery subgroup did not significantly differ. The pretreatment NLR was not predictive of prognosis when stage IV gastric cancer patients received palliative surgery. However, there were only two studies in the surgery group and three in the palliative chemotherapy group, and fewer included articles might have caused heterogeneity when we pooled the effect sizes. Hence, more attention should be focused on the predictive role of the NLR for late-stage gastric cancer in evaluating the prognosis of different treatments.

Studies of other tumors together with our study demonstrate that an elevated NLR plays an important role in predicting prognosis before treatment. However, the optimal cutoff value for NLR in predicting the prognosis of gastric cancer remains unclear. The cutoff values in our analysis ranged from 1.44 to 5.00, and they were determined by receiver operating characteristic curves, by the median value of all patients, or on the basis of previous studies, such as a score of 5.00. To establish a suitable cutoff value, we performed meta-regression and subgroup analyses with quartiles of the cutoff values (2.20, 3.00, and 4.00). The role of elevated NLR in predicting prognosis differed significantly among the four subgroups. In Subgroups 1 and 2 and Subgroups 3 and 4, the pooled HR was similar, which suggests that the HRs were almost the same when the cutoff values were set as the first two subgroups and the last two subgroups. The pooled HRs in Subgroups 3 and 4 were higher than those in Subgroups 1 and 2. From the results above, we thought that the predictive prognosis ability of the NLR might be slightly influenced by cutoff values when the range was from 1.44 to 5.00. We also found that when the cutoff value was set at 3.00, the results from original articles that used 3.00 as a cutoff value might be more stable and close to each other. However, in a study of 1028 patients, Shimada et al. [32] reported that an NLR of 4.00 appeared to be more useful than a cutoff value of 3.00, which was similar to our study. However, in our Subgroup 4, there were two studies that reported no significant difference with a cutoff value of 5 in multivariate analysis. The negative results of included articles in Subgroup 4 that may lead to the pooled result trend to be close to the result of Subgroup 3. Hence, we thought it may be a key point for performing a study of the NLR to define or help clarify an appropriate cutoff when the variation is wide. More attention should focus on the choice and comparison of cutoff values during analysis of the NLR in the future studies.

A previous meta-analysis evaluated the predictive role of the NLR for OS and DFS for gastric cancer [50]. Our study differed in several ways. Firstly, this study included eight more articles, which makes the results more powerful and robust. With the larger sample size, elevated NLR may reflect poor outcome in western and eastern countries. Secondly, we found that the NLR was higher in late-stage compared with early-stage gastric cancer. We discussed the predictive role of NLR in stage III and IV gastric cancer using rational and robust subgroups. Finally, this study explored suitable cutoff values for NLR for evaluating the prognosis of gastric cancer.

There were some limitations to our meta-analysis. First, all included articles were retrospective studies, and the level of evidence was not high enough. In addition, original articles supplied only summarized but not individualized data, which may have increased the heterogeneity of the articles. Second, not all studies supplied data for all analyses; thus, the results may be slightly influenced due to the limited number of included articles, particularly for the analysis of tumor differentiation and CEA. Third, sample size was analyzed as a potential source of heterogeneity. In the subgroup with fewer samples, heterogeneity was not significant. However, in the subgroup with more samples, significant heterogeneity was observed. Although the subgroup with fewer samples had no significant heterogeneity, studies including more samples might provide more robust results. For heterogeneity, sensitivity analysis could not provide additional information to address this limitation. Finally, several articles reported HRs, which, from the multivariate analysis and results, demonstrated no significant difference. These results might have been caused by other markers such as Glasgow score and CRP which may have a similar function as the NLR and influenced the analysis. We also aimed to address the confounding factors by sensitivity analysis, but we could not find a statistically significant result. More well-designed and high-quality multicenter clinical trials are required.

## 5. Conclusions

The presented meta-analysis demonstrated that pretreatment NLRs play a significant role in predicting the prognosis of gastric cancer, particularly for late-stage gastric cancer. Increased cutoff values of NLR may reflect prognosis as a biomarker better than the decreased values in gastric cancer. The ability of NLR to evaluate the prognosis of patients may be used in the future. Whether these findings can be used to adjust treatment decisions remains uncertain and is an area for further research.

## Figures and Tables

**Table 1:** Characteristics of included studies.

Name	Year	Country	Patients (female/male)	Age (range)	Treatment	Follow-up (month)	TMN (I/II/III/IV)	Tumor size^a^	CEA^b^	Tumor differentiation (well/poor)	Cutoff value^c^	Number of elevated NLR
Mohri et al. [18]	2014	Japan	123 (38/85)	Median: 66 (18–94)	Resection + chemotherapy	9.3	0/0/0/123	NA	NA	45/78	3.1	118
Jiang et al. [13]	2014	China	377 (124/253)	Median: 64 (25–80)	Resection + chemotherapy	42	37/99/241/0	140/237 (5 cm)	NA	97/280	1.44	309
Cho et al. [20]	2014	Korea	268 (93/175)	Mean: 55.4	Chemotherapy	11.3	0/0/0/268	NA	NA	95/173	3	138
Graziosi et al. [19]	2015	Italy	156 (92/64)	Median: 74 (39–91)	Resection + chemotherapy	23	42/29/62/23	NA	NA	NA	2.3	80
Aurello et al. [21]	2014	Italy	102 (40/62)	Median: 69	Resection	96	34/15/35/18	NA	NA	NA	5	28
El Aziz [22]	2014	Egypt	70 (23/47)	Median: 53 (30–70)	Resection	NA	0/0/49/21	NA	NA	NA	3	40
Lee et al. [23]	2013	Korea	174 (60/114)	Median: 55 (22–74)	Resection + Chemotherapy	14.9	7/22/41/101	NA	58/118	NA	3	62
Lee et al. [24]	2013	Korea	220 (71/149)	Mean: 57 (23–89)	Resection	NA	120/35/62/3	59/161	22/195	NA	2.15	56
Jin et al. [25]	2013	China	46 (10/36)	Median: 60 (37–77)	Resection + chemotherapy	NA	0/0/40/6	NA	NA	15/31	2.5	20
Dirican et al. [26]	2013	Turkey	236 (74/162)	Median: 58 (30–86)	Resection + chemotherapy	NA	6/20/105/105	NA	NA	NA	3.8	89
Wang et al. [14]	2012	China	324 (99/225)	NA	Resection + chemotherapy	39.9	0/0/324/0	158/168 (5 cm)	NA	NA	5	11
Kunisaki et al. [27]	2012	Japan	83 (26/57)	Mean: 67.7 (37–91)	Resection + chemotherapy	14.5	0/0/22/61	10/73 (5 cm)	NA	35/48	5	18
Kim and Choi [28]	2012	Korea	93 (36/57)	NA	Resection + chemotherapy	NA	44/16/33/0	60/33 (5 cm)	NA	44/49	1.8	36
Jeong et al. [29]	2012	Korea	104 (35/69)	Median: 52.5 (28–82)	Chemotherapy	11.9	0/0/0/104	NA	NA	27/75	3	55
Jung et al. [30]	2011	Korea	293 (100/193)	Median: 63 (21–96)	Resection + chemotherapy	27.2	0/0/143/150	NA	NA	73/220	2	155
Ubukata et al. [31]	2010	Japan	157 (51/106)	Mean: 65.27 (29–84)	Resection	NA	45/30/39/43	42/115	NA	58/99	5	70
Shimada et al. [32]	2010	Japan	1028 (319/709)	Median: 65 (26–89)	Resection	23	584/132/153/159	NA	NA	521/507	4	127
Mohri et al. [33]	2010	Japan	357 (112/245)	Median: 63.4 (32–87)	Resection	68	232/57/68/0	NA	NA	198/159	2.2	130
Yamanaka et al. [34]	2007	Japan	1220 (351/869)	NA	Chemotherapy	15.6	0/0/0/1220	NA	NA	NA	2.5	644

^a^Tumor size *⩾* cutoff value/tumor size < cutoff value; ^b^CEA *⩾* cutoff value/CEA < cutoff value; ^c^the cutoff value of NLR; NLR: neutrophil to lymphocyte ratio; NA: not applicable; TNM: tumor node metastasis stage; CEA: carcinoembryonic antigen.

**Table 2:** Quality assessment of included studies based on the Newcastle-Ottawa scales.

Name	A	B	C	D	E	F	G	H	Scroe
Mohri et al. [18]	*∗*	*∗*	*∗*	*∗*	*∗*	*∗*		*∗*	7
Jiang et al. [13]	*∗*	*∗*	*∗*	*∗*	*∗*	*∗*	*∗*	*∗*	8
Cho et al. [20]	*∗*	*∗*	*∗*	*∗*	*∗*	*∗*	*∗*		7
Graziosi et al. [19]	*∗*	*∗*	*∗*	*∗*		*∗*	*∗*	*∗*	7
Aurello et al. [21]	*∗*		*∗*	*∗*		*∗*	*∗*		5
El Aziz [22]	*∗*	*∗*	*∗*	*∗*		*∗*	*∗*		6
Lee et al. [23]			*∗*	*∗*		*∗*	*∗*		4
Lee et al. [24]			*∗*	*∗*		*∗*			3
Jin et al. [25]	*∗*	*∗*	*∗*	*∗*		*∗*	*∗*		6
Dirican et al. [26]	*∗*	*∗*	*∗*	*∗*		*∗*	*∗*		6
Wang et al. [14]	*∗*	*∗*	*∗*	*∗*	*∗*	*∗*	*∗*	*∗*	8
Kunisaki et al. [27]	*∗*	*∗*	*∗*	*∗*		*∗*	*∗*		6
Kim and Choi [28]			*∗*	*∗*	*∗*	*∗*	*∗*		5
Jeong et al. [29]	*∗*	*∗*	*∗*	*∗*	*∗*	*∗*	*∗*	*∗*	8
Jung et al. [30]	*∗*	*∗*	*∗*	*∗*		*∗*	*∗*		6
Ubukata et al. [31]	*∗*	*∗*	*∗*	*∗*	*∗*	*∗*	*∗*		7
Shimada et al. [32]	*∗*	*∗*	*∗*	*∗*	*∗*	*∗*	*∗*		7
Mohri et al. [33]	*∗*	*∗*	*∗*	*∗*		*∗*	*∗*		6
Yamanaka et al. [34]			*∗*	*∗*		*∗*	*∗*	*∗*	5

A: representativeness of the exposed cohort; B: selection of the nonexposed cohort; C: ascertainment of exposure; D: demonstration that outcome of interest was not present at start of study; E: comparability of cohorts on the basis of the design or analysis; F: assessment of outcome; G: follow-up long enough for outcomes to occur; H: adequacy of follow-up of cohorts.

**Table 3:** Summary of the meta analysis results.

Analysis	*N*	References	Fixed-effect model		Random-effect model		Heterogeneity		Meta regression
			HR (95% CI)	*p*	HR (95% CI)	*p*	*I* ^2^	*p*	*p*
Subgroup analysis for OS									
Subgroup: treatments									
Surgery	12	[13, 14, 18, 19, 21, 22, 24, 25, 28, 30–32]	2.01 (1.71–2.37)	<0.001	2.11 (1.72–2.57)	<0.001	26%	0.19	0.207
Chemotherapy	4	[20, 23, 29, 34]	1.95 (1.83–2.08)	<0.001	1.84 (1.48–2.28)	<0.001	86%	<0.001	
Mutlitherapy	3	[26, 27, 33]	2.39 (1.84–3.11)	<0.001	2.39 (1.84–3.11)	<0.001	1%	0.37	
Subgroup: region									
Western	3	[19, 21, 26]	2.26 (1.74–2.94)	<0.001	2.10 (1.42–3.10)	<0.001	44%	0.17	0.543
Eastern	16	[13, 14, 18, 20, 22–25, 27–34]	1.96 (1.85–2.08)	<0.001	1.96 (1.71–2.24)	<0.001	56%	0.004	
Subgroup: sample size									
Sample size ≥ 200	9	[13, 14, 20, 24, 26, 30, 32–34]	1.69 (1.53–1.86)	<0.001	1.82 (1.55–2.13)	<0.001	45%	0.07	0.034
Sample size < 200	10	[18, 19, 21–23, 25, 27–29, 31]	2.15 (2.00–2.31)	<0.001	2.15 (2.00–2.31)	<0.001	0%	0.43	
Subgroup: cutoff value									
(1) Cutoff ≤ 2.2	5	[13, 24, 28, 30, 33]	1.80 (1.43–2.26)	<0.001	1.80 (1.43–2.26)	<0.001	0%	0.43	0.112
(2) 2.2 < cutoff ≤ 3	7	[19, 20, 22, 23, 25, 29, 34]	1.96 (1.84–2.08)	<0.001	1.88 (1.56–2.26)	<0.001	0%	0.47	
(3) 3 < cutoff ≤ 4	3	[18, 26, 32]	2.32 (1.85–2.89)	<0.001	2.31 (1.81–2.94)	<0.001	41%	0.13	
(4) 4 < cutoff ≤ 5	4	[14, 21, 27, 31]	2.27 (1.59–3.26)	<0.001	2.36 (1.38–4.03)	0.002	54%	0.09	
Subgroup: stage IV									
Resection	2	[30, 32]	1.75 (1.30–2.36)	<0.001	1.73 (0.85–3.54)	0.13	83%	0.02	
Chemotherapy	3	[18, 20, 26, 29, 34]	1.94 (1.81–2.07)	<0.001	1.83 (1.49–2.24)	<0.001	90%	<0.001	

Clinicopathological parameters			OR (95% CI)	*p*	OR (95% CI)	*p*	*I* ^2^	*p*	

TNM stage (I + II vs. III + IV)	4	[13, 19, 24, 31]	2.59 (1.91–3.50)	<0.001	2.76 (1.36–5.61)	0.005	80%	0.002	
Tumor differentiation (well versus poor)	3	[13, 20, 30]	1.05 (0.77–1.43)	0.75	1.05 (0.77–1.44)	0.74	0%	0.38	
CEA (<5 ng mL^−1^ versus ≥5 ng mL^−1^)	2	[23, 24]	1.43 (0.64–3.21)	0.38	1.31 (0.77–2.25)	0.32	52%	0.15	
